# Transmission of Superoscillations

**DOI:** 10.1038/s41598-020-62018-7

**Published:** 2020-04-03

**Authors:** S. Zarkovsky, Y. Ben-Ezra, M. Schwartz

**Affiliations:** 1 Faculty of Electrical Engineering, Holon Institute of Technology (HIT), 52 Golomb St., Holon, 5810201 Israel; 2 School of Physics and Astronomy, Raymond and Beverley Faculty of Exact Sciences, Tel Aviv University, Tel Aviv, 69978 Israel

**Keywords:** Electrical and electronic engineering, Applied physics

## Abstract

It is widely accepted that a signal bandlimited by *σ* cannot oscillate at higher frequencies. The phenomenon of superoscillation provides a refutation of that quite general belief. Temporal superoscillations have been rarely demonstrated and are mostly treated as a mathematical curiosity. In the present article we demonstrate experimentally for the first time to our best knowledge, the transmission of superoscillating signals through commercial low pass filters. The experimental system used for the demonstration is described, providing the insight into the transmission of superoscillations, or super-narrow pulses. Thus, while the phenomenon may seem rather esoteric, a very simple system is used for our demonstration.

## Introduction

**Figure 1:**
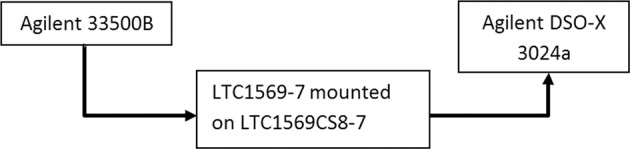
experimental setup.

**Figure 2:**
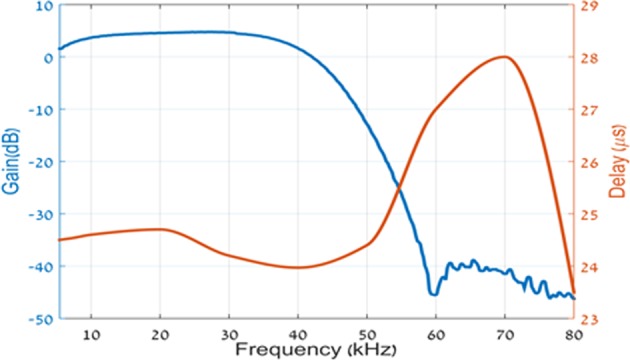
Frequency response, gain(blue) and group delay (red) of the analog circuit (10^*th*^ order low pass filter LTC1569-6).

We start our discussion, rather unconventionally, by presenting some simple yet intriguing experimental results, even before any formal introduction. In Fig. 1 we present a schematic description of our experimental setup. It consists of an arbitrary shape waveform generator (Agilent 33500B), a low pass filter (LTC1569-6) and an oscilloscope (DSO-X 3024 A). All are off the shelf commercial not oversophisticated components. In Fig. 2 we show the transmission characteristics of our low pass filter.

**Figure 3:**
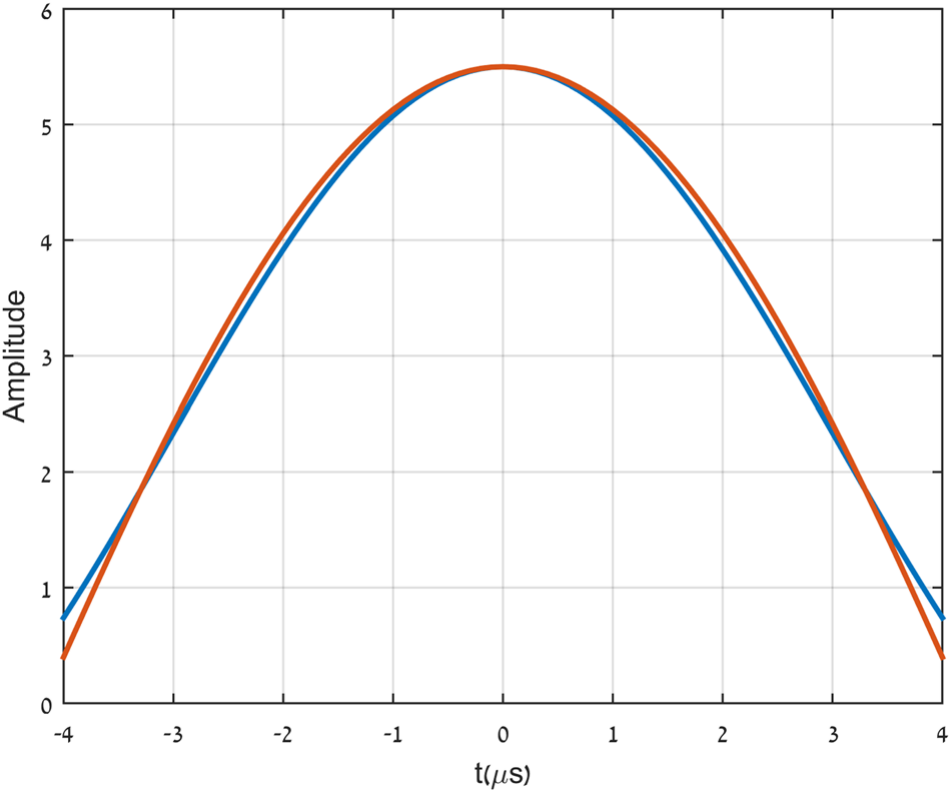
The two input pulses.

**Figure 4:**
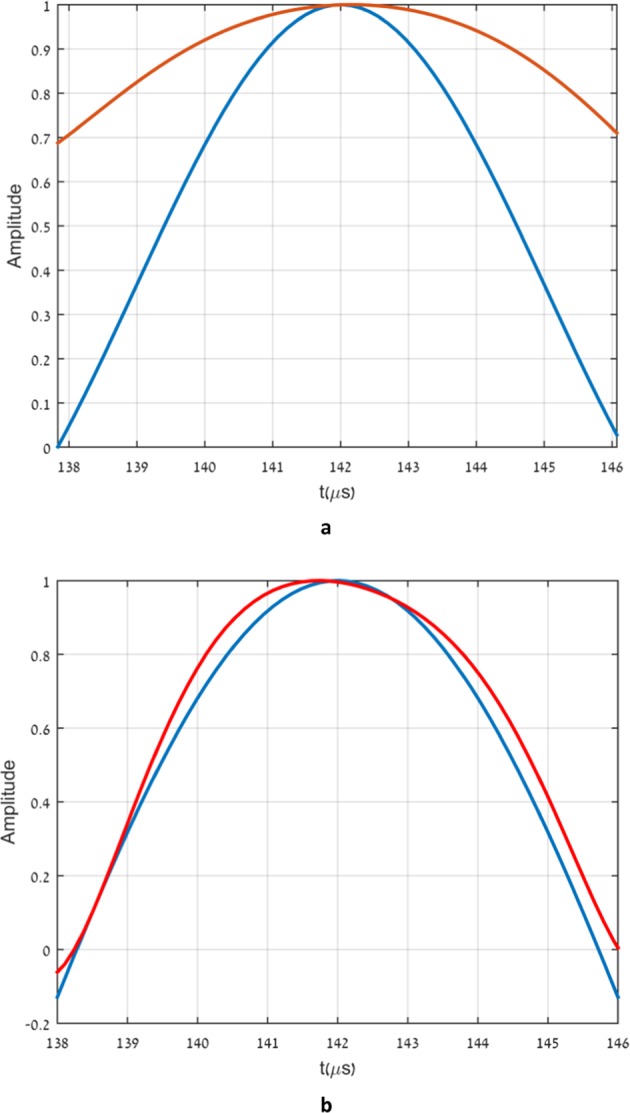
(**a**) - the generic output vs the input pulse. (**b**) - The special transmitted pulse vs the corresponding input pulse. The output pulses as well as the input pulses were normalized for clear comparison.

In Fig. 3 we show two almost identical pulses. The pulses are fractions of two different signals, generated by the arbitrary waveform generator. The two signals are then transmitted through the low pass filter described in Fig. 2. A very natural expectation is that both pulses will not be transmitted as is but will be considerably broadened to respect the limitations imposed by the low pass filter. The shape and duration of both pulses seem to indicate that both input signals contain frequencies above the effective band limit of the filter. Indeed we see in Fig. 4a the expected generic transmission. The transmitted pulse (in red) is broadened considerably relative to the input pulse (in blue) due to the loss of the high frequency components. In Fig. 4b we see that the second, almost identical pulse, after being transmitted through the same filter (in red) is almost not broadened at all relative to the input signal (in blue).The input pulses are relatively not noisy. The output pulses, on the other hand, are rather noisy. The output pulses depicted in Fig. 4 are actually polynomial fits to the noisy data recorded by the oscilloscope.

The difference between the two cases cannot be but in the way in which the two pulses are complemented to generate the two longer signals. Thus, not only the rise and fall time of the pulse are relevant to its transmission but also features of the signal outside the pulse itself!!. The transmission shown in Fig. 4a is the generic case, which is typical of the overwhelming majority of ways in which the pulse is complemented to the longer signal. The transmission shown in Fig. 4b is an extremely rare case, where the duration of the transmitted pulse is almost unaffected by the low pass filter. The blue and red curves in Fig. 4 correspond to the output and input pulses respectively.

The input pulses in Fig. 3 are almost identical but the way in which the signal is complemented is quite different. The input signal, corresponding to the output pulse in Fig. 4a, is a periodic pulse with period 2*π*∕*σ* defined by:

1 $${f}_{n}(t)=\mathop{\sum }\limits_{l=-n}^{n}{e}^{il\sigma t},$$

with *nσ* the highest frequency component. The input signal, corresponding to the output pulse in Fig. 4b is given by:

2 $${g}_{n}^{m}(t)={f}_{m}(t)+\mathop{\sum }\limits_{l=-n}^{-m-1}{h}_{n}^{m}(l,\sigma t)+\mathop{\sum }\limits_{l=m+1}^{n}{h}_{n}^{m}(l,\sigma t)$$

where *mσ* grazes the effective band limit of the low pass filter. The functions ${h}_{n}^{m}(l,\sigma t)$ are required to obey the following: (a)Each of those function are band limited by the filter bandwidth. (b)Within the width of *f*_*n*_(*t*), ${h}_{n}^{m}(l,\sigma t)$ approximates *e*^*ilσt*^ to a required accuracy. By construction, ${g}_{n}^{m}(t)$ is limited within the transmission band of the low pass filter and is made to adhere to *f*_*n*_(*t*) at small values of *σt*. The functions ${h}_{n}^{m}(l,\sigma t)$, are band limited yet oscillate locally at a frequency higher than the band limit. Such signals are called superoscillations. Consequently, the highest frequency component present in ${g}_{n}^{m}(t)$ is only *mσ*.

## Theory, Experimental Results and Discussion

**Figure 5:**
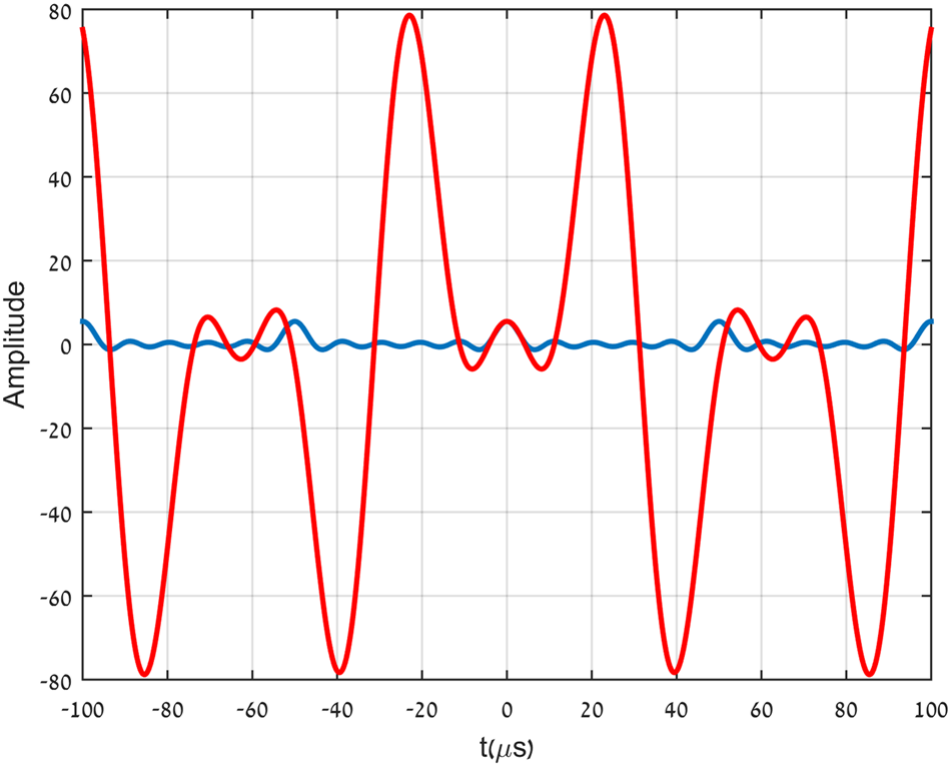
The blue curve is the full input signal, corresponding to the output pulse in Fig. 4a. The entire input signal corresponding to output pulse in Fig. 4b is depicted by the red curve. Fig. 3 consists of a zoom in around the origin of Fig. 5.

To understand the concept of superoscillation, consider a band limited signal-it could be optical, electrical or acoustic, it does not really matter. Although the signal will have a power spectrum that spans a certain frequency range, an analysis of the signal over particular time intervals might show, however, local oscillations with a frequency outside the frequency range of the signal. These are called superoscillations. A number of examples have been given for such signals^1–4^ with suggested applications in various fields such as signal processing^5–8^ and quantum mechanics^1,3,9,10^. The concept of superoscillations is also most useful in optics, where it is intimately related to super resolution^11–16^. In contrast to what is going on in the other fields mentioned, where the application of superoscillations is more a theoretical study of the possibilities, the application to optics is in a much more advanced phase of practical experimental study^17–22^. The present more practical application of superoscillations in optics is based, however, (with the exception of ref. ^18^) on spatial structures on scales smaller than the wave length of the light interacting with those structures. This results in beams narrower than allowed by the Rayleigh-Abbe diffraction limit . The phenomenon of superoscillation is very exciting and seems to suggest many possible and beautiful applications. Yet the applications are still quite limited. The reason is that in a sense, the widely accepted lore, that frequencies higher than the band limit cannot be observed even locally in a band limited signal, is not entirely false. The fact is that superoscillations are possible but they come at a high price. It is well known that the superoscillations exist in limited time intervals and that the amplitude of the superoscillations is extremely small compared to typical values of the amplitude in the non-superoscillating parts of the signal. The ratio between the two depends on the ratio of superoscillation frequency to the band limit frequency but it depends also much stronger (exponentially) on the number of superoscillations in those intervals^23^ and is also sensitive to noise^24,25^. This fact and the dynamic range if the oscilloscope, limits the range of band limited functions ${h}_{n}^{m}(l,\sigma t)$ that can be used to construct the superoscillatory pulse in Fig. 5.

The signal described by the red curve in Fig. 5 is based on an analytic expression given first by Aharonov, Popescu and Rohrlich^1^:

3 $${f}_{k}(vt,\omega /v)={\left(\cos (\frac{vt}{k})+i\frac{\omega }{v}\sin (\frac{vt}{k})\right)}^{k}.$$

It is well known that *f*_*k*_(*vt*, *ω*/*v*) ≈ *e*^*iωt*^ for $vt < \sqrt{\varepsilon k/[{(\omega /v)}^{2}-1]}$, where *ε* is the allowed tolerance, regardless of the fact that it is easily verified that *f*_*k*_(*vt*, *ω*/*v*) is band limited by *v* and that *ω*/*v* may be arbitrarily larger than 1^2^.This is an example of a superoscillating signal. It is band limited by *v* yet for arbitrary long time, depending on *εk*, it oscillates with a frequency *ω*, higher than the band limit. We chose:

4 $${h}_{n}^{m}(l,\sigma t)={f}_{n}(m\sigma t,l/m)$$

The red curve is obtained using ${g}_{n}^{m}(t)$ with *n* = 5 and *m* = 2. Figs. 3 and 4 become now very clear. The signal ${g}_{5}^{2}(t)$ is obtained by replacing in *f*_5_(*t*) the components with frequencies above the filter band limit (40*kHz*) by the corresponding APR signals, which superoscillate locally near the origin with the same frequency. This explains why the signals ${g}_{5}^{2}(t)$ and *f*_5_(*t*) are very close to one another in the vicinity of the origin, as seen in Fig. 3. Next, it is clear from equation (1) that the constituent frequencies of *f*_5_(*t*) are of the form 20*nkHz*, with *n* = 0,...,5, Thus, the frequencies 60, 80 and 100*kHz*, are removed by the low pass filter and the output signal is broadened (Fig. 4a). On the other hand, all the real constituent frequencies of ${g}_{5}^{2}(t)$ are of the form 8*nkHz*, with *n* = 0, …, 5, and thus below the effective band limit. Thus, the necessary condition for the transmission of all frequency components is fulfilled. This however, is not yet sufficient for the central pulse to pass the filter only slightly distorted. The dependence of the group delay of the filter on frequency (Fig. 2), at frequencies below the band limit, is of crucial importance for that. Fortunately, the nonlinearity of the group delay vs. frequency of the filter in the relevant frequency range (Fig. 2), is not large enough to distort the transmitted pulse significantly.

**Figure 6:**
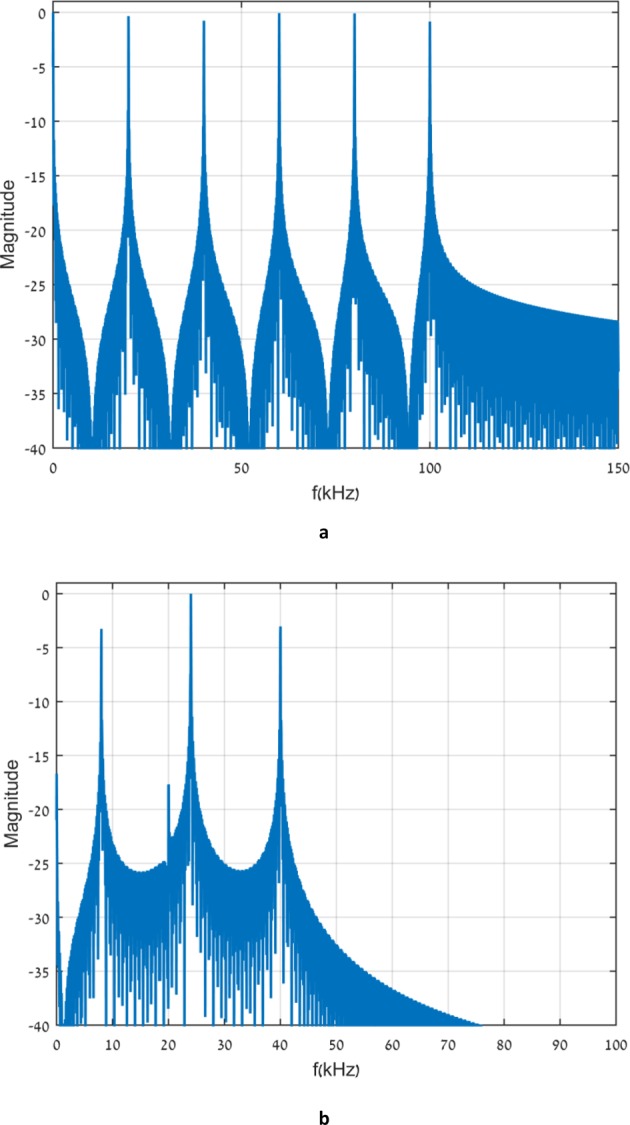
Fourier transform of transmitted signals where (**a,b**) corresponds to *f*_5_(*t*) and ${g}_{5}^{2}(t)$ respectively. Note that the Fourier transform in (**b**) seems to miss two frequencies. This is not the case but may look so because on the right hand side of Eq. (2) not all the frequency components appear with the same weight,contrary to the right hand side of Eq. (1).

Our Explanations above rely on Eqs. (1)–(3), which give the form of the idealized signals. The real input signals must be different due to the finite duration of the signal and might affect our arguments above. Therefore, we present in Fig. 6 Fourier transforms of the two time restricted signals corresponding to the input signals *f*_5_(*t*) and ${g}_{5}^{2}(t)$. The Fourier transform of the realization of *f*_5_(*t*) exhibits six distinct peaks which are to a good approximation located at 20*nkHz* and are more or less of the same height. A lot of weight exists at frequencies above the band limit. Thus, the output signal is broadened as discussed above. The Fourier transform of ${g}_{5}^{2}(t)$ is mostly concentrated below 40*kHz*. This is consistent, of course, with the fact that the output pulse is not broadened. As mentioned above this would not have sufficed for the approximate shape recovery of the central pulse.

It is important to note, that we could have chosen on the right hand side of Eq. (4) other analytic superoscillating^26–29^ signals instead of the APR signals^2^ to obtain similar results. In fact, it could be expected that using instead the APR signals, yield optimized signals^23^ would result in better looking input superoscillating pulses. As can be seen, however, from the above it is not necessary for our demonstration. Off the shelf equipment and the very convenient APR functions are sufficient to make our point. Namely, it is actually possible to pass through a low pass filter “too narrow” pulses and it does not involve a high level of sophistication.

## Impact of phase variations on super oscillatory signal

Since superoscillation is a delicate destructive interference phenomenon, it is important to understand the sensitivity of our results to random phase shifts. First, it is clear that in the pulse ${g}_{n}^{m}(t)$, the contribution most sensitive to random phase shifts is *f*_*k*_(*mσt*, *n*/*m*). Its general form (which includes also other superoscillating signals) is given by:

5 $${f}_{k}(m\sigma t,n/m)=\mathop{\sum }\limits_{j=0}^{k}A\left(j,k,\frac{n}{m}\right){e}^{i\frac{2j-k}{k}m\sigma t}$$

Introducing random phase shifts results in:

6 $${f}_{k}\left\{\phi ;m\sigma t,\frac{n}{m}\right\}=\mathop{\sum }\limits_{j=0}^{k}\left(j,k,\frac{n}{m}\right){e}^{m\sigma ti\frac{2j-k}{k}+i{\phi }_{j}}$$

We assume that the *ϕ*_*j*_’s are not correlated, that the average of *ϕ*_*j*_,  < *ϕ*_*j*_ > = 0, $< {\phi }_{j}^{2} > =\delta \ll 1$ and therefore $< exp(i{\phi }_{j}) > =1-\frac{1}{2}\delta$. We will get the idea of how small *δ* should be by taking two averages. The first average is just

7 $$\left\langle {f}_{k}\left\{\phi ;m\sigma t,\frac{n}{m}\right\}\right\rangle =\left(1-\frac{1}{2}\delta \right){f}_{k}(m\sigma t,n/m)$$

Thus, for small *mσt* the average still approximates *e*^*inσt*^, consider next:

8 $$\left\langle {\left|{f}_{k}\left\{\phi ;m\sigma t,\frac{n}{m}\right\}\right|}^{2}\right\rangle ={\left(1-\frac{1}{2}\delta \right)}^{2}{\left|{f}_{k}(m\sigma t,n/m)\right|}^{2}+\left[1-{\left(1-\frac{1}{2}\delta \right)}^{2}\right]\mathop{\sum }\limits_{j=0}^{k}{\left|A\left(j,k,\frac{n}{m}\right)\right|}^{2}$$

Thus the precise condition on *δ*, that ensure that the random phase shifts do not destroy the local high frequency of the superoscillation is

9 $$\mathop{\sum }\limits_{j=0}^{k}{\left|A\left(j,k,\frac{n}{m}\right)\right|}^{2}\delta \ll 1$$

which is considerably more stringent than just *δ* ≪ 1

## Summary

We have presented a generic way of constructing interesting super- narrow structures, which include super positions of pure superoscillations (A different, quite interesting way of fitting a general polynomial over a finite range by superoscillating function had been presented in^30^). We have considered the transmission of electrical signals of that nature through low pass filters, relative to which those structures are "too narrow” . We have demonstrated the possibility of transmission of such narrow structures in the lab using rather basic equipment. We showed that the faithful transmission can be achieved with real world components even though strict superoscillation is only a mathematical idealization, We have also obtained the sensitivity of superoscillations to random phase shifts of the constituent Fourier components. We expect to come back to the fascinating phenomenon of superoscillation in the very near future. It seems to open many interesting directions of research^31,32^.
